# The Modelled Raindrop Size Distribution of Skudai, Peninsular Malaysia, Using Exponential and Lognormal Distributions

**DOI:** 10.1155/2014/361703

**Published:** 2014-07-08

**Authors:** Mahadi Lawan Yakubu, Zulkifli Yusop, Fadhilah Yusof

**Affiliations:** ^1^Institute of Environmental and Water Resources Management, Universiti Teknologi Malaysia, 81310 Skudai, Johor, Malaysia; ^2^Department of Civil Engineering, Faculty of Engineering, Kano University of Science and Technology, 713101 Wudil, Kano, Nigeria; ^3^Department of Mathematical Sciences, Faculty of Science, Universiti Teknologi Malaysia, 81310 Skudai, Johor, Malaysia

## Abstract

This paper presents the modelled raindrop size parameters in Skudai region of the Johor Bahru, western Malaysia. Presently, there is no model to forecast the characteristics of DSD in Malaysia, and this has an underpinning implication on wet weather pollution predictions. The climate of Skudai exhibits local variability in regional scale. This study established five different parametric expressions describing the rain rate of Skudai; these models are idiosyncratic to the climate of the region. Sophisticated equipment that converts sound to a relevant raindrop diameter is often too expensive and its cost sometimes overrides its attractiveness. In this study, a physical low-cost method was used to record the DSD of the study area. The Kaplan-Meier method was used to test the aptness of the data to exponential and lognormal distributions, which were subsequently used to formulate the parameterisation of the distributions. This research abrogates the concept of exclusive occurrence of convective storm in tropical regions and presented a new insight into their concurrence appearance.

## 1. Introduction

Rain event is normally an expression of varied composition of raindrops diameters as a function of their volumetric diameters per unit volume of space [1]. Raindrop size distribution (DSD) defines the variation in the composition of different raindrop sizes (diameters) within a storm and could be used as a tool for classifying rain events [2, 3]. The rainfalls in temperate climatic zones are composed of small to average size drops in contrast to tropical zones, which are composed of higher proportions of larger raindrops, typically from short-duration high-intensity storms [4]. In the field of communication, rainfalls are categorised into three categories: the drizzle, showers, and thunderstorms. In communication field, DSD is one of the major sources of error in any DSD model because of its temporal and spatial variation between geoclimatic regions [5]. However, in the field of hydrology, rainfalls are categorised into two categories based on their physical processes: the convective and the stratiform. Majority of the established DSD functions used disdrometer recordings [5–9]. However, disdrometer was found to be biased towards larger raindrops by underestimating smaller drops [10].

Although studies of DSD were carried out in other tropical regions [3, 6, 7, 11–15], to the best of our knowledge, there was no formulation or model representing the characteristics of DSD in Malaysia. The climate of Malaysia is more local than regional [16] and it is distinctive in its characteristics and often cannot be subjected to similarity to other regions. The previous research of DSD using data obtained from Kuala Lumpur by Lam [17] focused on investigating the dependence of the rain attenuation on the DSD and finding the key raindrop diameter for computing specific rain attenuation, rather than establishing the relationships of DSD in the region with defined equation's fittings and coefficients. This lack of established relations could limit further studies in the region. Therefore, the aim of this study is to establish DSD relations based on the region's rainfall characteristics that could provide a tool for modelling urban hydrological process, flood appraisal, and prediction of rain attenuation with ease.

Techniques used to measure raindrop diameter, and its distribution, can broadly be classified into two: the automatic equipment and the manual methods. The absorbent paper method devised by Lowe [18] and documented by Wiesner [19], the flour pellet method developed by Bentley [20] which was modified by Laws and Parsons [21], and the oil immersion method fashioned by Eigel and Moore [22] are examples of the latter category, while disdrometer, an electromechanical feeler that translates the momentum of falling raindrop into electrical recordable pulses developed by Joss and Waldvogel [23], and photography method developed by Jones [24] and advanced into the Optical-Spectropluviometer are examples of the former category. Most researchers use acoustic instrument to measure raindrop diameter and its distribution [5, 14, 25–27]. However, this equipment is known to underestimate small raindrop's diameters [10]. In this research, we used unbiased method to bin each raindrop size appropriately to its size by sieving method.

A raindrop breaks into smaller diameters when it reaches its limiting size of about 5 mm to 8 mm [28–30]. The breaking up of raindrop size after reaching a threshold value of 5 mm suggests that DSD follows the form of an exponential function at higher intensity. Lenard [31] was the first to study the breakup of water drops based on separation of electric charges principle. Raindrop breaks because of the induced aerodynamics of resisting air acting on the centre mass of the drop and other reasons such as collision between drops. A raindrop exhibits a complicated shape; however, it forms an almost perfect sphere at small diameters less than 1.25 mm and is flattened at the bottom due to resisting air pressure forming an oblate spheroid shape at larger diameters [29, 32, 33]. At about 10 mm, the hydrodynamic forces overcome the internal binding forces causing air forces to cause a break of the drop into smaller sizes [12]. Recently, Villermaux and Bossa [30] investigated both the shape of the drops' sizes and their distribution and concluded that the DSD parameters are related to the dynamics of the whole spectrum of sizes observed in rain. They further highlighted that topological withering in raindrops from big to smaller unwavering sizes is attained within a much shorter timescale than the typical collision time between the drops.

## 2. Materials and Methods

### 2.1. Study Area

**Figure 1:**
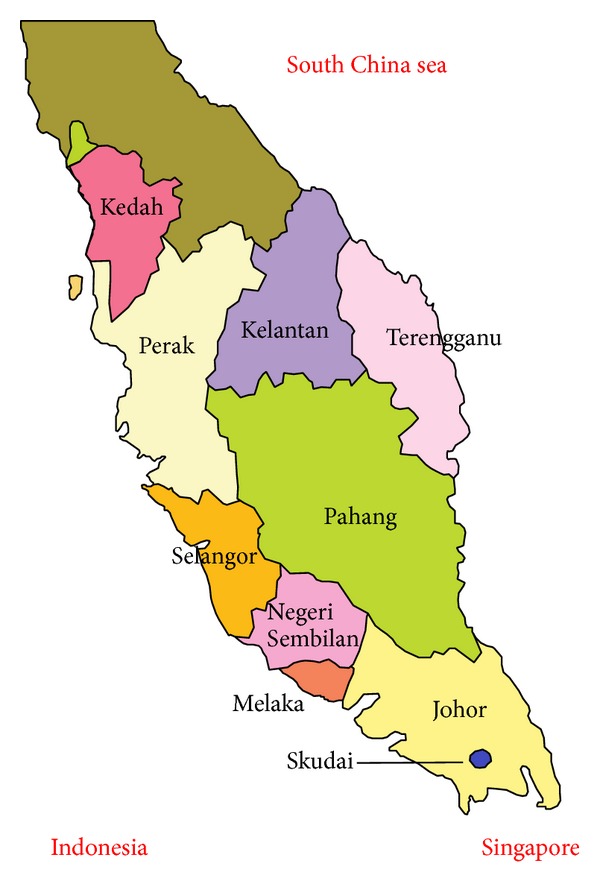
Location map of the study area.

The map of the study area is shown in Figure 1. Skudai is located within west Peninsular Malaysia which boarders Thailand to the north and stretched southward to Singapore. It lies between 6°45′ and 1°20′N latitudes and 99°40′ and 104°20′E longitudes. Skudai lies within the Intertropical Convergence Zone (ITCZ) which results in elevated temperature and high humidity [34]. Skudai experiences an average of 2000 to 2500 mm rainfall depth annually [35]. The study area is characterised by a monthly regular uniform rain distribution dominated by convective storms [34, 35].

### 2.2. Experimental

A highly sensitive tipping bucket (model RG3-M) that can record up to 3,200 rainfall events was equipped with an event data logger and mounted on coordinate 103°38′39.5′′E 1°33′41.6′′N inside Universiti Teknologi Malaysia (UTM) away from any lateral obstructions. Before its use, the rain gauge was recalibrated according to manufacturer's instruction to ±1% accuracy. Intensities were recorded on one-minute basis. The exact time and temperature were also recorded, which were used in the estimation of the rain intensity and the drop diameter, respectively. The equipment was checked every fortnight to ensure its proper functioning and to ensure it was on upright position of 1.8 m above ground level.

At selected storms, the flour pellet method was used to trap raindrops as they approach the ground. A 3 mm thick flour was spread on a 0.15 m^2^ rectangular tray and briefly exposed to seventeen different rain intensities for about 3–5 seconds, depending upon the strength of the intensity, such that enough raindrops would have been trapped. The profile of the sampled storms and the number of samples in each storm was presented in Table 1.

**Figure 2:**
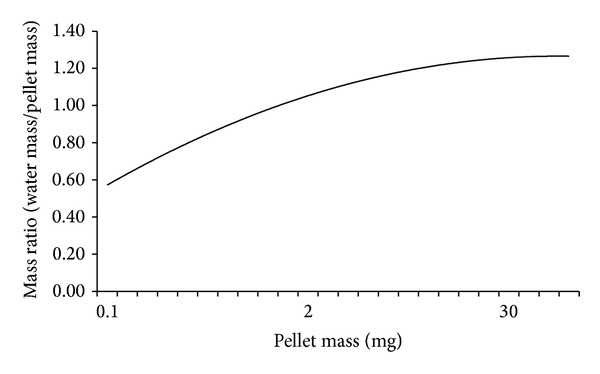
Hudson [4] calibration curve.

The formed capsules were marked and immediately transported to a laboratory and oven dried using automated universal oven (Memmert, model 16.1) for 12 hrs at 105°C. The flour capsules were divided into different size fractions according to BS 812-103.1:1985 method for determination of particle size distribution. Thus, the oven dried flour samples were poured into 300 mm diameter standard sieves. The sieves were then stacked in decreasing size (6.30, 5.00, 4.47, 2.36, 2.00, 1.18, and 0.60 mm) and secured onto a mechanical shaker. The contents were allowed to vibrate for 10 min. The retained pellets were carefully dislodged and emptied into a preweighted stainless steel container with the help of a handheld brush. The weight of each particle size fraction retained in any given sieve was obtained using a weighing balance accurate to 0.001 g. A total of 720859 capsules were counted and measured. The drop diameter for each sieve class in a given sample was then calculated by converting the weight of the flour capsule into an appropriate raindrop diameter using the Hudson [4] calibration curve shown in Figure 2. Six raindrop diameter classes were obtained (1.95–2.65, 2.67–3.67, 3.68–5.81, 5.82–6.00, 6.10–7.02, and >7.03 mm) based on the corresponding sieve sizes used.

### 2.3. Parameterization of Drop Size Distributions

Raindrop distribution can be estimated from exponential equation suggested by Marshall and Palmer [36] of the form shown in

(1) $$\begin{array}{c} N\left(D\right)=N_{0}e^{-\lambda D}, \end{array}$$

where *D* is the drop diameter (mm), *N*_0_ is a constant (8000 m^3^ mm^−1^) that corresponds to *N*(*D* = 0), and *λ* is a parameter (mm^−1^) that depends on the rainfall intensity (*I*) as shown (2). The parameters *λ* and *N*_0_ for different climatic regions can be determined experimentally [14]. Consider

(2) $$\begin{array}{c} \lambda =cI^{-d}, \end{array}$$

where *c* = 4.1 and *d* = 0.21. It is well established in the literature that the DSD follows gamma distribution [14, 37] which is an improved form of the Marshall and Palmer [36] equation proposed by Ulbrich [38] of the form shown in

(3) $$\begin{array}{c} N\left(D\right)=N_{0}D^{\mu }e^{-\lambda D}, \end{array}$$

where *μ* is a dimensionless shape coefficient.

The basic difference between (1) proposed by Marshall and Palmer [36] and (3) by Ulbrich [38] is the number of parameters. The lognormal model, shown in the form of (4), has advantages over other functions, because all of its three parameters have physical significance and the parameters have linear relations to the moment of DSD [5]

(4) $$\begin{array}{c} N\left(D\right)=\frac{N_{D}}{\sigma D\sqrt{2\Pi }}\text{EXP}\left[-\frac{{\left(\ln D-\mu \right)}^{2}}{2\sigma ²}\right], \end{array}$$

where *N*(*D*) is the number of densities (m^−3^ mm^−1^), *N*_*D*_ is the drops count (m^−3^), and *μ* and *σ* are the logarithmized mean and standard deviation of the drop diameters and could be obtained from (5) and (6), respectively. Consider

(5) $$\begin{array}{c} \sigma =\sqrt{\ln\left(1+\frac{v_{r}}{m²}\right)}, \end{array}$$

(6) $$\begin{array}{c} \mu =\ln\left(\frac{m²}{\sqrt{v_{r}+m²}}\right); \end{array}$$

*m* and *v*_*r*_ are the mean and variance of nonlogarithmized values in the measured samples obtained from method of moment. The third, fourth, and sixth moments were used for estimating the parameters.

## 3. Results and Discussion

**Figure 3:**
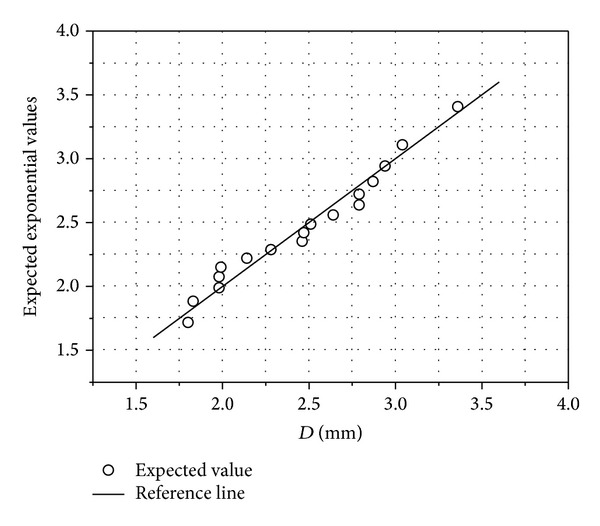
Exponential Q-Q plot.

**Figure 4:**
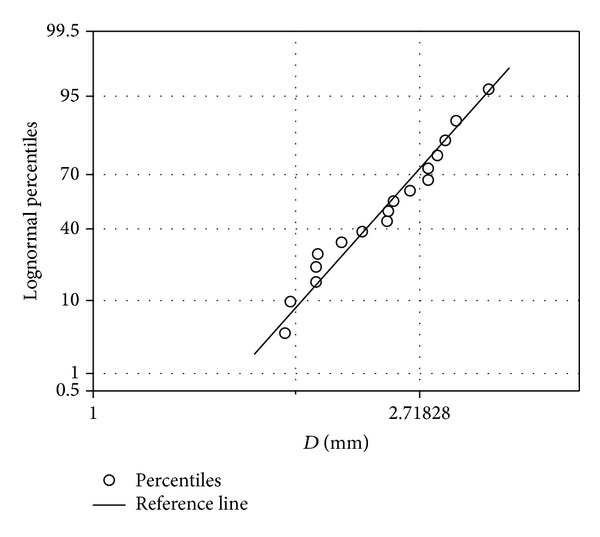
Lognormal probability test plot.

The quantile-quantile (Q-Q) and probability plots were respectively used to test whether our study data follows the exponential and lognormal distributions. The Kaplan-Meier method was used for the survival analyses. The Q-Q and probability plots for the median drop diameter for each intensity were presented in Figures 3 and 4, respectively. The figures indicated the veracity of our study data to suit the lognormal and exponential distributions.  Figure 3 presents the exponential Q-Q plot showing the studied raindrop sizes values on *x*-axis and their expected values on *y*-axis, while Figure 4 is the lognormal probability plot which is a plot showing the observed cumulative percentage of raindrop sizes on *x*-axis and their expected cumulative percentiles on *y*-axis.

The difference between the two figures is the representation of the values in percentiles in Figure 4 instead of their real values as shown in Figure 3. The closer the scattered points are to the expected value line the stronger the indication that it follows the given distribution. Therefore, subject to this survival test, the method of moment was used to estimate the real mean and standard deviation of (5) and (6) which were used in defining scale and shape parameters in (4).

The exponential *N*_0_ parameter in (1) was estimated from regression analysis of our data after eight iterations and was found to be 7627 counts. The *λ* parameter depends on intensity [3, 14]. The *λ*-rain rate relationship of Skudai climatic region was established from our data by individually fitting the intensity with the corresponding *λ* value from (2). This resulted in the following:

(7) $$\begin{array}{c} \lambda =3.3I^{-0.25}. \end{array}$$

Brodie and Rosewell [1] summarised *N*_0_ obtained from different studies. They noted that *N*_0_ varies from 1400 counts for thunderstorm to a maximum value of 30000 for drizzle and 7000 for widespread rain. Coutinho and Tomás [3] reported *N*_0_ counts higher than 11500 and a least of 3900 counts from their studies which was composed of similar upper and lower rain intensities considered in this study. *N*_0_ values can be used to classify storm classes based on their metrological nature. According to Waldvogel [2], *N*_0_ less than 2000 signifies convective storm (where the weighted composition of DSD leans towards disposition of larger drops than smaller drops), while *N*_0_ in excess of 20000 implies stratiform storm (where the continuum balance between larger and smaller drops swings towards small drops). The storms considered in this study have spatial representation. The storms are composed of rain intensities less than 35 mm h^−1^ with only two of the intensities higher than 70 mm h^−1^. The *N*_0_ obtained from this study supported the description of the study area rainfall pattern by Zin et al. [39] and Shamsudin and Dan'azumi [34]. Likewise, the summarised result from Brodie and Rosewell [1] recorded the *c* coefficient in (2) between 3.0 and 5.8 while the *d* coefficients range between 0.20 and 0.21 units.

**Figure 5:**
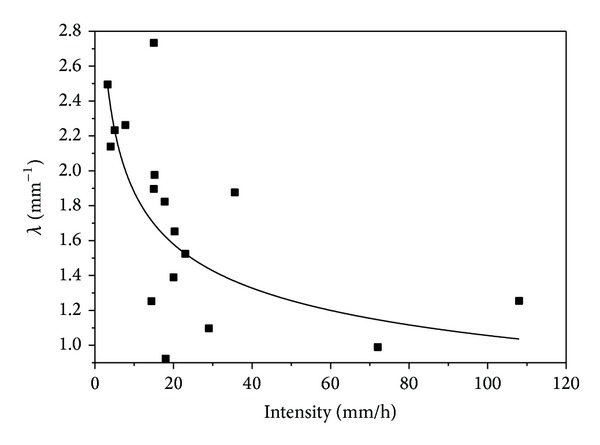
*λ*-intensity relationship.

Figure 5 shows the modelled rain rate parameter for the seventeen different rain intensities; the coefficient of determination obtained from the fit was 0.56. *λ* decreases with increasing rain intensity. The *λ*-intensity relationship suggests that smaller rain intensities are composed of smaller but higher counts of raindrops, while higher intensities have the least collection of raindrops number but with larger drops diameters.

**Figure 6:**
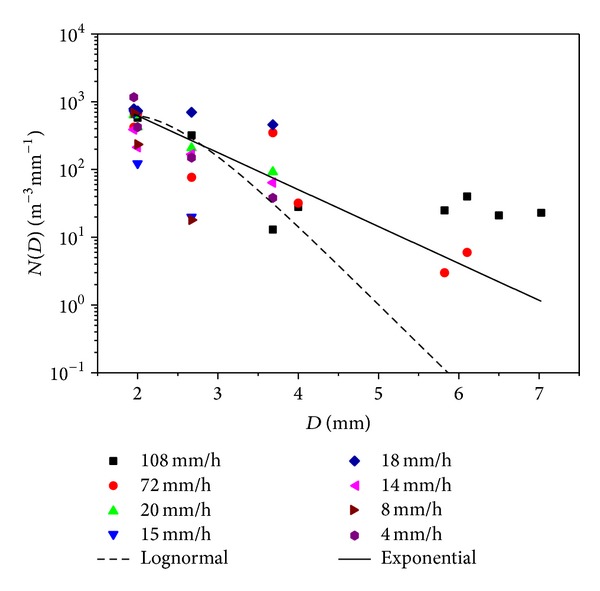
Exponential and lognormal DSD of Skudai.

Figure 6 presents the characteristics of Skudai DSD. The fitted lines conform to (1) and (4), respectively, for exponential and lognormal models with *R* ^2^ of 0.72 and 0.64, respectively. The exponential and lognormal models in Figure 6 represented the range of data obtained from this study, with the lognormal model tending to underestimate the DSD of smaller intensities at drop diameters smaller than 4 mm, while the exponential model predisposed to the bigger diameters at moderate to higher intensities.

Both the lognormal and the exponential models show consistent trends at drop diameter of less than 3.3 mm. The result also shows that higher rain intensities are composed of larger proportions of raindrop diameters than lighter intensities. The rain intensity in Skudai is considerably composed of raindrop diameters of less than 4 mm in large part. Taking into cognisance the *N*_0_ count obtained from this study, the study area could be characterised by combined convective and stratiform widespread uniform rainfall. Similar occurrence of convective storm and stratiform in tropical regions has been reported in geographical regions of western equatorial Pacific and northern Australia [40].

**Figure 7:**
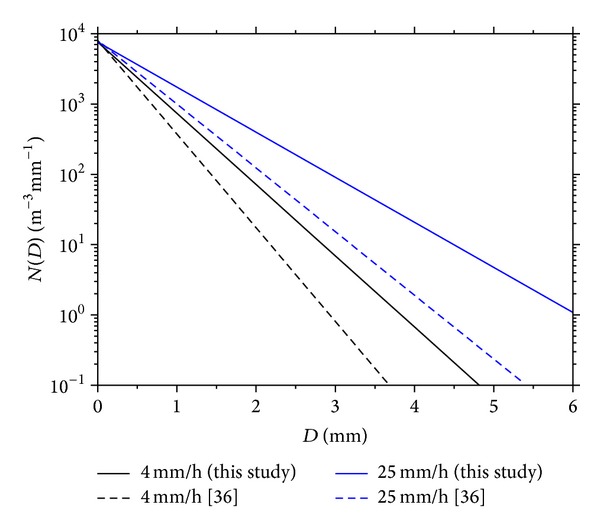
Modelled drop size distribution using exponential distribution density function.

The exponential DSD model obtained from this study is compared with Marshall and Palmer [36] model at intensities of 4 mm h^−1^ and 25 mm h^−1^ as shown in Figure 7. The results of the two models are in agreement with maximum divergence at higher raindrop diameter. Thus, the models compare more than 70% at drop diameters of 4 mm or less. This is quite expected as the maximum drop diameter considered by Marshall and Palmer [36] is in the order of 4 mm. But both models approached a common value as the rain diameters approach zero. The model converges at *N*_0_ = 7627 corresponding to *N*  (*D* = 0) for all rain intensities considered in this study.

The lognormal parameters of the DSD obtained in (4) were related to the intensity using regression analysis on the data. *N*_*D*_, *σ*, and *μ* are known to relate to the intensity [5]. These parameters relate to the intensity of the region as presented in

(8) $$\begin{array}{c} N_{D}=763I^{0.69},\\ \sigma =0.31\ln\left(I\right)-0.44,\\ \mu =2.34-2.00\ln\left(I\right). \end{array}$$

**Figure 8:**
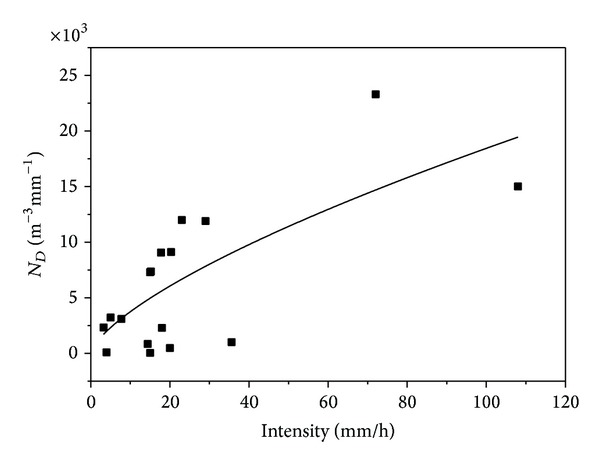
Drops count-intensity relationship.

**Figure 9:**
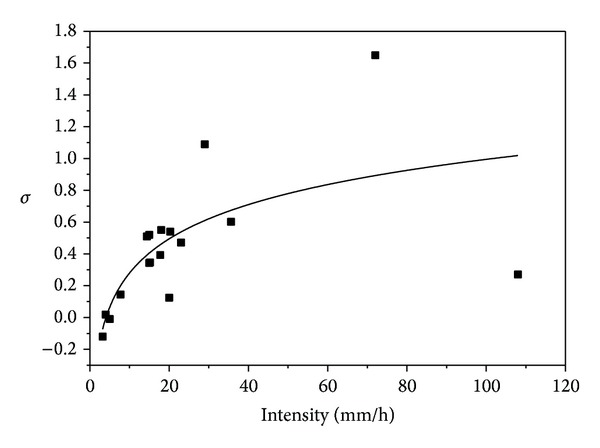
Logarithmized standard deviation-intensity relationship.

**Figure 10:**
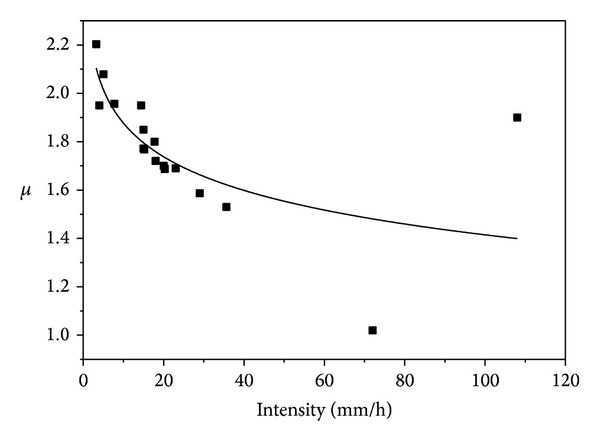
Logarithmized mean-intensity relationship.

These modelled parameters and their relationship with intensity are presented in Figures 8–10.

Figures 8 and 9 indicated that *N*_*D*_ and *σ* increase with increasing rain rate while Figure 10 suggests decrease of *μ* with increasing intensity. Timothy et al. [5] suggested that the three modelled lognormal parameters are not sufficient to describe the rain rate based on their data. The result of this study, however, indicated that the *σ* and *μ* relationships in Figures 8 and 9 are sufficient to describe rain rate especially at intensity of less than 40 mm h^−1^. But the *N*_*D*_ relationship with intensity presented in Figure 8 suggests that the *N*_*D*_ depends not only on the rain rate but also on other climatic parameters like the rain type.

A differentiation between the convective and stratiform storms is very valuable in the tropics and in mid-scopes in the warm season of other geographies, as condensation peaks during the latent heat liberation in troposphere zones of stratiform precipitation. Therefore, a combined model of the exponential and the lognormal distributions could describe tropical storm of both convective and stratiform storms in more appropriate manner than using a single model.

## 4. Conclusions

Five different parametric expressions describing the rain rate of Skudai were established from this study. The exponential and the lognormal models were used to describe the DSD of the study area. The parameters of these models were empirically instituted from the experimental result using regression analysis on the data. The modelled rain rate and the drop count per unit volume of rain obtained from this study infer that the study area experiences uniform precipitation. This research also demonstrated that the convective storm in the tropical region of Skudai occurred concurrently with stratiform storm.

The advantage of using more than one model to predict storm behaviour has been put forward in this study. The results of the two models are in agreement, with a maximum divergence at a higher raindrop diameter. The lognormal model tends to underestimate the DSD of lighter rain intensities at drop diameters smaller than 4 mm, while the exponential model was predisposed to the bigger diameters at moderate to severe rain intensities. The *λ*-intensity relationship suggested that lighter rain intensities were composed of smaller but higher counts of raindrops, while higher intensities have the least collection of the raindrop's numbers but with larger drop's diameters. The result from this study indicated that the *σ* and *μ* relationships from the lognormal model are sufficient to describe rain rate, especially at intensities of less than 40 mm h^−1^. But the *N*_*D*_ relationship with intensity suggested that the drop counts depend not only upon the rain rate but also on other climatic parameters.

## Figures and Tables

**Table 1:** Sampled storm profile.

Sampling date	Duration (minutes)	Average storm intensity (mm hr^−1^)	Number of samples
09/10/12	120	35	2
01/11/12	60	12	7
27/6/13	33	12	1
16/7/13	47	16	1
18/08/13	22	29	1
24/08/13	15	8	1
26/08/13	110	65	1
03/10/13	56	32	1
05/10/13	13	21	1
12/10/13	34	23	1
